# Post-shift recovery sleep duration and its association with daytime sleepiness among night-shift nurses: a repeated-measures observational study

**DOI:** 10.3389/fpubh.2026.1835119

**Published:** 2026-06-04

**Authors:** Ye Feng, Liangzhu Feng, Wen Shi

**Affiliations:** 1 Department of Anesthesiology, Tangdu Hospital, The Fourth Military Medical University, Xi'an, China; 2 School of Physical Education & Sports Science, South China Normal University, Guangzhou, China

**Keywords:** daytime sleepiness, Epworth Sleepiness Scale, fatigue management, night-shift nurses, sleep duration

## Abstract

**Objective:**

To examine the association between post-shift recovery sleep duration and daytime sleepiness among night-shift nurses.

**Methods:**

A repeated-measures observational study among night-shift nurses in a tertiary hospital. Data were collected between September and November 2025. Post-shift recovery sleep duration, defined as sleep obtained within 24 h after completing a night shift, was categorized as 0 h, <2 h, 2–4 h, or ≥4 h. Daytime sleepiness was assessed using the Epworth Sleepiness Scale (ESS). Linear mixed-effects models with random intercepts were applied to evaluate associations while controlling for sex, working unit, night-shift workload, caffeine intake during night shifts, and insomnia severity.

**Results:**

Recovery sleep duration was associated with daytime sleepiness across sleep-duration categories. Nurses reporting 2–4 h of recovery sleep had the highest ESS scores compared with those reporting no recovery sleep (ES, ±95% *CI*: 0.5, ±0.29; *p* < 0.001), <2 h (2.76, ±0.23; *p* < 0.001) or ≥4 h (1.62, ±0.25; *p* < 0.001). This pattern was generally consistent across clinical units, although differences were attenuated in high-acuity settings such as emergency, intensive care, and operating room units.

**Conclusions:**

Post-shift recovery sleep duration was associated with daytime sleepiness among night-shift nurses, with intermediate recovery sleep duration (2–4 h) showing the highest ESS scores. Because recovery sleep duration was self-reported and the study design limits causal inference, these findings should be interpreted as associations rather than evidence of causality. Fatigue risk management strategies should consider not only sleep duration but also the quality and context of post-shift recovery.

## Introduction

Night-shift nursing is essential for maintaining continuous 24-h patient care, yet working through the biological night challenges the human circadian system and sleep–wake regulation. Night work commonly produces circadian misalignment and shortened sleep because daytime sleep after a night shift occurs at an adverse circadian phase, when the drive for wakefulness is relatively high and the ability to obtain consolidated, restorative sleep is reduced (1). In addition, competing daytime demands (e.g., family and social responsibilities) can further curtail post-shift sleep opportunity, contributing to cumulative sleep debt and impaired alertness (1).

Excessive daytime sleepiness (EDS) is a highly prevalent and consequential outcome among nurses and other shift-working healthcare staff. EDS is generally defined as a tendency to fall asleep unintentionally during the daytime, reflecting an increased propensity for sleep in situations where wakefulness is expected (2). A recent systematic review and meta-analysis reported that EDS affects nearly one-third of nurses globally, underscoring the scale of the problem and the need for effective fatigue-risk mitigation strategies (3). Empirical studies in real-world clinical contexts also link elevated sleepiness to safety risks. For example, among night-shift nurses and midwives in an NHS hospital trust, 28% met an ESS threshold indicative of abnormal daytime sleepiness, and higher ESS scores were associated with adverse safety outcomes such as drowsy driving and near-miss incidents when commuting after night shifts (4). Beyond individual safety, sleepiness in clinical staff has implications for work performance, quality of care, and patient safety, reinforcing the importance of identifying modifiable sleep-related factors in night-shift schedules (4).

Shift work sleep disorder (SWSD)—characterized by insomnia and/or excessive sleepiness temporally associated with a work schedule that overlaps usual sleep time—provides a clinical framework for understanding chronic maladaptation to shift work. Large epidemiologic research in nurses has shown that symptoms indicative of SWSD can be common, with prevalence estimates varying substantially depending on assessment approach and schedule characteristics (5). Importantly, SWSD and EDS are not solely determined by the presence of night work; they are shaped by work–rest patterns (e.g., quick returns, number of night shifts), individual vulnerability, and sleep obtained between and after shifts (5). Among the potential countermeasures, strategic napping during night shifts has been widely studied. A systematic review concluded that night-shift napping generally decreases sleepiness and improves sleep-related performance, although short periods of sleep inertia may occur immediately after waking (6). More recently, quasi-experimental evidence suggests that taking a nap or even a structured break immediately after a night shift may support fatigue recovery and influence subsequent sleep episodes, highlighting the broader concept of “post-work recovery” as part of fatigue risk management (1).

Post-shift recovery sleep is a particularly relevant and modifiable component of off-duty time because it is the most immediate opportunity to reduce homeostatic sleep pressure accrued during the night shift. However, recovery sleep after night work is often fragmented or curtailed, and its relationship with daytime sleepiness may be non-linear due to competing processes such as circadian timing, sleep inertia, and incomplete dissipation of sleep debt (7). Additionally, nurses' work environments differ markedly (e.g., emergency, intensive care, operating room, general wards), which may shape both post-shift sleep behavior and subjective sleepiness through differences in workload intensity, stress, and opportunities for rest (1). These factors support the need for unit-sensitive, real-world evidence that focuses specifically on recovery sleep duration after night shifts.

Therefore, this study aimed to examine the association between post-shift recovery sleep duration (sleep obtained within 24 h after completing a night shift) and daytime sleepiness among night-shift nurses using repeated questionnaire measurements in a tertiary hospital setting. By categorizing recovery sleep into clinically interpretable intervals and modeling within-individual variation while accounting for key work- and sleep-related covariates, this study seeks to clarify whether specific ranges of post-shift sleep are linked to higher or lower daytime sleepiness and to inform practical recommendations for fatigue risk mitigation in night-shift nursing.

## Methods

### Study design and participants

This study was designed as a repeated-measures observational study, conducted among nurses working night shifts in a tertiary hospital. A convenience sampling strategy was used to recruit participants from different clinical units within the hospital. Data were collected through a self-administered questionnaire between September and November 2025. Registered nurses were eligible for inclusion if they: (1) were currently engaged in clinical nursing practice, (2) were performing night-shift work during the study period, and (3) had completed at least one night shift in the past 7 days. Nurses who were not working night shifts during the study period were excluded. Participation was voluntary. All participants were informed of the study purpose and procedures and provided informed consent prior to completing the questionnaire. The questionnaire was completed anonymously. Ethics committee approval of this study was gained from the local university (Approval No.: SCNU-SPT-2024-070).

### Data collection

Data were collected using a structured questionnaire distributed online. The questionnaire was administered in three survey rounds, with each round separated by approximately 3–4 weeks, and repeated responses from the same participants were recorded. A total of 223, 198, and 179 valid responses were obtained in survey rounds 1, 2, and 3, respectively. Among the 238 unique participants, 24 completed one survey round, 66 completed two rounds, and 148 completed all three rounds. Of the 623 questionnaires distributed, 600 valid responses were obtained, yielding a valid response rate of 96.3%. A total of 600 valid responses were obtained from 238 unique participants, with each participant contributing between 1 and 3 observations. Repeat entries from the same participants were tracked using unique anonymous identifiers generated by the survey system, enabling linkage of responses across survey rounds. Responses with duplicate identifiers and inconsistent information were reviewed and excluded where necessary to ensure data integrity. To ensure data quality, questionnaires with missing key variables or obvious logical inconsistencies were excluded from the final analysis.

### Measures

#### Post-shift recovery sleep duration

Post-shift recovery sleep duration was the primary predictor in this study. Participants were asked to report the average duration of sleep obtained within 24 h after completing a night shift, including daytime sleep. Recovery sleep duration was categorized into four groups: 0 h, < 2 h, 2–4 h, and ≥4 h. These categories were selected according to the distribution of the study data and prior evidence showing that post-night-shift sleep among nurses often falls within short-to-moderate daytime sleep ranges, commonly around 3–6 h (8). In addition, nap and shift-work literature indicates that shorter sleep episodes and longer daytime sleep periods may have different effects on sleepiness, recovery, and sleep inertia (6). Importantly, the recovery sleep assessed in this study represents daytime sleep obtained within 24 h after a night shift, rather than total daily or nocturnal sleep duration. Therefore, the selected categories do not correspond to recommended sleep durations for adults (e.g., ≥7 h per night), but instead reflect realistic patterns of post-shift recovery sleep observed in shift-working populations. This categorization was used to examine differences in daytime sleepiness across clinically relevant post-shift recovery sleep-duration groups. Sleep quality and the timing of recovery sleep within the 24-h post-shift period were not specifically assessed in the present study.

#### Daytime sleepiness

Daytime sleepiness was assessed using the Epworth Sleepiness Scale (ESS), a widely used and validated self-report questionnaire designed to measure general daytime sleep propensity. The ESS consists of eight items assessing the likelihood of dozing in common daily situations, with each item scored from 0 to 3, yielding a total score ranging from 0 to 24. Higher scores indicate greater daytime sleepiness, and an ESS score of 10 or higher was used to define excessive daytime sleepiness. The validity and reliability of the ESS have been well established, and the scale has been widely applied in clinical populations, occupational groups, and shift-working populations, including nurses and other healthcare workers (9–12). The Epworth Sleepiness Scale primarily reflects general daytime sleepiness rather than shift-specific or post-awakening sleepiness, and was used in this study as an indicator of overall sleep propensity. Therefore, ESS scores in the present study should be interpreted as reflecting overall daytime sleep propensity rather than immediate post-shift sleepiness.

#### Covariates

Demographic and work-related variables were collected, including age, sex, body mass index (BMI), working unit, years of work experience, number of night shifts during the past 7 days, and number of consecutive night shifts. Sleep-related and lifestyle variables included average sleep duration on non-night-shift days, caffeine intake during night shifts, and insomnia severity assessed using the Insomnia Severity Index (ISI). The ISI is a widely used and validated self-report questionnaire that assesses the severity and impact of insomnia symptoms over the past 2 weeks. Total ISI scores range from 0 to 28, with higher scores indicating more severe insomnia. The reliability and validity of the ISI have been well established, and the scale has been extensively used in clinical populations, occupational groups, and shift-working populations, including nurses and other healthcare workers (12, 13). For the purpose of this study, ISI scores were dichotomized, with a cut-off score of ≥8 used to define the presence of insomnia symptoms, consistent with established thresholds in the literature.

#### Statistical analysis

Linear mixed-effects models were performed using PROC GLIMMIX in the University Edition of the Statistical Analysis System (SAS Studio version 3.6). Daytime sleepiness score was treated as the dependent variable. A random intercept for nurse identity was included in all models to account for within-individual correlation due to repeated measurements. This modeling approach allowed the inclusion of all available repeated observations while appropriately accounting for intra-individual dependence. Covariates were selected *a priori* based on theoretical relevance and prior literature to minimize the risk of overfitting (31, 32). Fixed effects included post-shift recovery sleep duration, sex, working unit, number of night shifts during the past 7 days, number of consecutive night shifts, caffeine intake during night shifts, and insomnia severity assessed using the Insomnia Severity Index (ISI). Sex and working unit were included as categorical variables. Caffeine intake during night shifts and insomnia status based on the ISI were included as binary variables, while the number of night shifts during the past 7 days and the number of consecutive night shifts were treated as ordinal or continuous variables, as appropriate. Model diagnostics were performed to assess the assumptions of the linear mixed-effects models. Normality of residuals and homogeneity of variance were evaluated using residual plots and Q–Q plots, and no substantial violations were observed.

The established models were used to estimate adjusted mean differences in daytime sleepiness across post-shift recovery sleep duration categories, while controlling for the effects of sex, working unit, number of night shifts during the past 7 days, number of consecutive night shifts, caffeine intake during night shifts, and ISI. Standardized effects were additionally interpreted using the following scale: < 0.2 trivial, ≥0.2 to < 0.6 small, ≥0.6 to < 1.2 moderate, ≥1.2 to < 2.0 large, and ≥2.0 very large. Standardized effects were calculated by dividing the adjusted mean differences by the between-subject standard deviation derived from the mixed-effects model. Effects were described based on the probability that the true effect was trivial or substantial, using qualitative likelihood terms (< 0.5% most unlikely, ≥0.5% to < 5% very unlikely, ≥5% to < 25% unlikely, ≥25% to < 75% possibly, ≥75% to < 95% likely, ≥95% to < 99.5% very likely, and ≥99.5% most likely), following the magnitude-based inference framework proposed by Hopkins (14). This magnitude-based inference approach was applied as a supplementary descriptive method alongside conventional statistical inference, and results were interpreted cautiously. Survey round was not included as a fixed effect because the primary focus of the analysis was on within-individual associations, and repeated measurements were accounted for by including a random intercept for participant identity.

## Result

**Figure 1:**
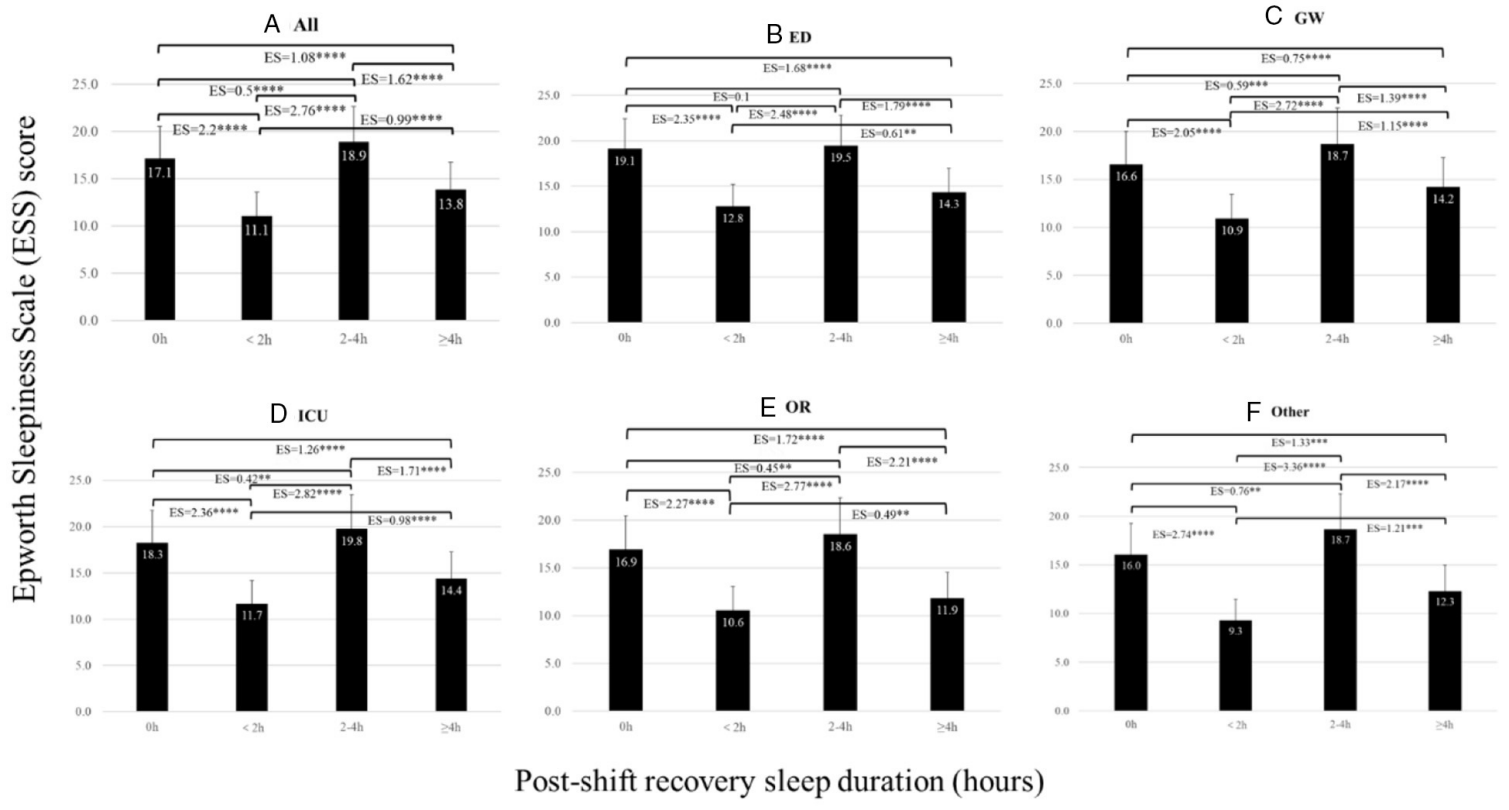
Differences in Epworth sleepiness scale (ESS) scores across post-shift recovery sleep duration categories among nurses. The number of asterisks indicates the likelihood of the magnitude of the true effect as follows: *possibly; **likely; ***very likely; ****most likely. Abbreviations: ED, emergency department; GW, general ward; ICU, intensive care unit; OR, operating room. **(A)** All. **(B)** ED. **(C)** GW. **(D)** ICU. **(E)** OR. **(F)** Other. The number of asterisks indicates the likelihood of the magnitude of the true effect as follows: ^*^ possibly; ^**^ likely; ^***^ very likely; ^****^ most likely.

The demographic characteristics of the participants are presented in Table 1, and descriptive characteristics stratified by post-shift recovery sleep duration are shown in Table 2. Table 2 presents unadjusted descriptive statistics, whereas Figure 1 shows adjusted means derived from mixed-effects models accounting for covariates. Figure 1 illustrates the differences in Epworth Sleepiness Scale (ESS) scores across categories of post-shift recovery sleep duration.

**Table 1:** Demographic characteristics of the study participants.

Characteristics	Male (*n* = 72)	Female (*n* = 528)
Age	30.5 ± 4.7	29.8 ± 5.7
Years-experience	8.8 ± 4.8	7.9 ± 5.9
BMI	23.9 ± 2.8	23.7 ± 3.2

**Table 2:** Participant characteristics stratified by post-shift recovery sleep duration.

Variable	0 h (*n* = 67)	< 2 h (*n* = 151)	2–4 h (*n* = 271)	≥4 h (*n* = 111)
ESS score, mean ±*SD*	15.30 ± 2.28	9.52 ± 2.90	17.34 ± 2.49	12.09 ± 3.03
Age, years	30.24 ± 5.41	29.56 ± 5.61	29.95 ± 5.79	30.23 ± 5.54
Years of experience	8.73 ± 5.72	7.74 ± 5.78	8.01 ± 5.97	8.39 ± 5.79
BMI, kg/m^2^	24.69 ± 3.27	23.53 ± 2.98	23.64 ± 3.17	23.71 ± 3.55
Night shifts (past 7 days)	2.46 ± 1.27	2.93 ± 1.40	2.73 ± 1.36	2.83 ± 1.40
Consecutive night shifts	1.36 ± 0.60	1.43 ± 0.61	1.47 ± 0.63	1.59 ± 0.68
Caffeine intake (cups/night shift)	1.70 ± 1.15	1.54 ± 1.14	1.42 ± 1.11	1.50 ± 1.20
Insomnia symptoms (ISI ≥ 8), *n* (%)	59 (88.1)	127 (84.1)	227 (83.8)	90 (81.1)
General ward (GW), *n* (%)	33 (49.3)	83 (55.0)	136 (50.2)	61 (55.0)
Emergency department (ED), *n* (%)	5 (7.5)	18 (11.9)	40 (14.8)	15 (13.5)
Intensive care unit (ICU), *n* (%)	10 (14.9)	19 (12.6)	41 (15.1)	19 (17.1)
Operating room (OR), *n* (%)	11 (16.4)	12 (7.9)	29 (10.7)	12 (10.8)
Other units, *n* (%)	8 (11.9)	19 (12.6)	25 (9.2)	4 (3.6)

Overall, ESS scores were highest among nurses reporting 2–4 h of recovery sleep. Compared with the 0-h group, ESS scores were higher in the 2–4 h group (ES, ±95% *CI*: 0.5, ±0.29; *p* < 0.001). ESS scores in the 2–4 h group were also higher than those in the < 2 h group (2.76, ±0.23; *p* < 0.001) and the ≥4 h group (1.62, ±0.25; *p* < 0.001). In addition, ESS scores in the ≥4 h group were higher than those in the < 2 h group (0.99, ±0.25; *p* < 0.001). Similar results were observed in the GW and Other units in Figures 1C, F. However, in the ED, ICU, and OR units in Figures 1B, D, E, ESS scores for a recovery sleep duration of 2–4 h were only higher than those for < 2 h (2.48, ±0.69; *p* < 0.001) and ≥4 h (1.62, ±0.71; *p* < 0.001).

## Discussion

The present study investigated post-shift recovery sleep obtained within 24 h after a night shift and its association with daytime sleepiness measured by the Epworth Sleepiness Scale (ESS) in a cohort of night-shift nurses with repeated questionnaire assessments. The central observation was that daytime sleepiness differed across recovery sleep-duration categories: recovery sleep of 2–4 h was associated with the highest ESS scores across units, whereas both shorter sleep (< 2 h or none) and longer sleep (≥4 h) were associated with lower sleepiness levels after adjustment for insomnia severity, night-shift burden, caffeine intake, sex, and unit. These findings may be clinically relevant because excessive daytime sleepiness is common in nursing populations globally and is linked to safety-critical outcomes, including drowsy driving and near-miss events after night work (3).

Much of the existing literature implicitly supports a monotonic narrative in which less sleep predicts greater sleepiness and fatigue in shift-working clinicians (15, 16); however, the present results suggest that, in the specific window of immediate post-night-shift recovery, “more” sleep may not necessarily correspond to “less” sleepiness. Epidemiologic and field studies in nurses consistently document curtailed and poor-quality sleep surrounding night shifts and show that sleepiness levels can be elevated in a substantial proportion of staff (4, 17). Yet these studies typically index total sleep over a day or sleep between shifts, rather than isolating recovery sleep immediately after a night shift. By focusing on the first post-shift 24 h, the present work aligns with a growing fatigue-risk perspective that emphasizes post-work recovery as a distinct and modifiable segment of the duty cycle, while suggesting that recovery sleep duration may capture heterogeneous physiological and behavioral states rather than representing a simple “dose” of recuperation (18, 19).

The elevated sleepiness observed in the 2–4 h category may be related to sleep-wake regulation models in which recovery sleep after night work is likely constrained by circadian misalignment and may be fragmented, lighter, and less restorative than nocturnal sleep (20). Daytime sleep following a night shift is obtained when circadian wake-promoting signals are relatively strong; consequently, an intermediate-length sleep episode might reduce homeostatic pressure sufficiently to permit awakening while not fully providing consolidated restorative sleep across multiple sleep cycles. Under this scenario, could awaken with residual sleep drive, yielding high subjective sleepiness even though some sleep has occurred (21). This interpretation also reconciles why the ≥4 h group was not uniformly “best”: if longer daytime sleep remains fragmented or is terminated at an unfavorable circadian phase, subjective sleepiness may remain elevated despite greater time in bed (22–24).

One possible explanation may involve the interplay between recovery sleep duration and sleep inertia. Experimental work on napping during night work demonstrates that post-sleep impairment can occur immediately after awakening, and that subjective sleepiness does not always track objective impairment in a straightforward way (25, 26). Although classic laboratory paradigms often emphasize short naps ( ≤ 60 min), the present field pattern could be consistent with the possibility that a 2–4 h recovery episode could more frequently include deep sleep and/or end in a sleep stage associated with pronounced inertia, particularly when awakening is externally constrained (family responsibilities, commuting, or required duties). In contrast, very short naps (< 2 h) may in some cases be associated with less severe post-awakening inertia, while longer sleep (≥4 h) could permit completion of additional sleep cycles, reducing the probability of awakening from deep sleep and improving perceived recovery. Importantly, because ESS reflects a general propensity to doze rather than momentary post-awakening sleepiness, the inertia account is likely partial rather than exhaustive; nevertheless, the literature supports the broader principle that timing and structure of sleep can modulate alertness outcomes in ways not captured by sleep duration alone (27–29).

An alternative, and epidemiologically plausible, interpretation is that the 2–4 h category may function as a marker for a subgroup experiencing the greatest physiological strain or the poorest recovery conditions rather than indicating a causal detrimental effect of that specific duration. Shift work disorder and shift-related insomnia symptoms are common in nursing, and are associated with work schedule characteristics, insufficient recovery between duties, and comorbid insomnia/anxiety features (5). Even with statistical adjustment for insomnia severity, residual confounding may be present: nurses who are most sleep-deprived, most stressed, or most physiologically hyperaroused may be more likely to report an intermediate amount of recovery sleep because they are unable to sustain longer consolidated daytime sleep, yet are also too fatigued to remain awake without attempting to sleep. In this framing, 2–4 h may represent “attempted recovery under constraint,” which aligns with field evidence that sleep between/after night shifts is often limited and disrupted in real healthcare settings (4). The non-linearity may therefore arise from a mixture of mechanisms—circadian constraints, sleep fragmentation, externally forced awakening, and reverse causation—rather than a single pathway.

Unit-level heterogeneity offers additional insight into the plausibility of these mechanisms. The persistence of the “2–4 h highest” pattern across units may suggest that circadian timing and physiological characteristics of daytime recovery sleep are broadly relevant to night-shift nursing regardless of specialty. At the same time, attenuated contrasts in higher-acuity settings (e.g., ED/ICU/OR) may reflect the possibility that workload intensity, stress exposure, and heightened autonomic arousal degrade sleep quality across all recovery-sleep durations, thereby compressing differences between categories. This interpretation accords with broader shift-work research indicating that shift schedules are associated with impaired sleep quality even when ESS differences are small or inconsistent, underscoring that sleepiness may reflect a complex blend of sleep quantity, sleep quality, and occupational strain (30).

From a fatigue-risk management standpoint, the findings may support a shift from purely quantitative messaging (“sleep more after nights”) toward guidance that recognizes the vulnerability of daytime recovery sleep to circadian and environmental constraints. The literature on nurse sleepiness underscores the magnitude of the problem and its safety implications, strengthening the case for organizational strategies that protect recovery opportunities and reduce hazardous outcomes such as drowsy driving (3). Practically, interventions may need to target not only duration but also recoverability—sleep environment control (light, noise), schedule design that minimizes quick returns, and education on managing awakening and transitions from sleep. The non-linear pattern specifically suggests that nurses obtaining an intermediate amount of recovery sleep may represent a high-risk phenotype for sleepiness who could be prioritized for targeted mitigation strategies.

Several limitations should be considered when interpreting the present findings relative to prior studies. Recovery sleep duration was self-reported and categorized, which may introduce misclassification and obscure meaningful variation in sleep timing, fragmentation, and sleep stage composition. In addition, the categorization of recovery sleep duration, although informed by prior literature and data distribution, remains somewhat arbitrary and may not fully capture clinically meaningful thresholds. ESS captures trait-like sleep propensity rather than acute sleepiness; thus, the observed associations likely reflect cumulative sleep debt and chronic circadian strain as much as immediate post-shift effects. Moreover, as assessed by the Epworth Sleepiness Scale, daytime sleepiness reflects general sleep propensity rather than shift-specific or post-awakening sleepiness, which may lead to a mismatch between the exposure (post-shift recovery sleep) and the outcome. This mismatch could potentially attenuate the observed associations. Importantly, key dimensions of sleep such as sleep timing (e.g., circadian phase of sleep) and sleep quality (e.g., fragmentation, subjective restfulness) were not comprehensively captured in the present dataset, limiting a more nuanced interpretation of recovery processes. In particular, the timing of recovery sleep within the post-shift 24-h period (e.g., immediate vs. delayed sleep following the night shift) may influence subsequent sleepiness, as suggested by prior research (1), and warrants further investigation. The cross-sectional design also limits causal inference and leaves open the possibility of reverse causation (greater sleepiness prompting sleep attempts of a particular length). Although a range of covariates was collected, only a subset was included in the final models based on theoretical relevance and prior literature, and residual confounding from unmeasured or excluded variables cannot be ruled out. However, the use of repeated assessments with a random-intercept mixed-effects approach strengthens internal validity by accounting for within-person correlation and reducing bias from stable individual differences, making the non-linear association less likely to be a simple artifact of between-person variability.

Future research would benefit from integrating objective measures (actigraphy or wearable-derived sleep staging where feasible) to quantify timing, fragmentation, and awakening context, enabling direct tests of competing explanations (e.g., inertia vs. constrained recovery). Pairing ESS with state sleepiness measures (e.g., Karolinska Sleepiness Scale) and performance indicators (e.g., psychomotor vigilance) would clarify whether the 2–4 h window primarily reflects acute post-awakening phenomena or a broader vulnerability to sleepiness. Given the established prevalence of excessive daytime sleepiness among nurses and its demonstrated safety relevance, such work has clear translational value for designing unit-sensitive, evidence-based recovery guidance for night-shift staff.

## Conclusion

This study found a clear non-linear association between post-shift recovery sleep duration and daytime sleepiness among night-shift nurses. Recovery sleep of 2–4 h was associated with the highest levels of daytime sleepiness, whereas both shorter (< 2 h or none) and longer (≥4 h) recovery sleep durations were associated with lower sleepiness levels after adjustment for relevant covariates. These findings suggest that intermediate-duration recovery sleep may represent a particularly vulnerable recovery state following night work. Optimizing post-shift recovery strategies should therefore consider not only sleep duration but also the broader context of recovery quality and work demands.

## Data Availability

The raw data supporting the conclusions of this article will be made available by the authors, without undue reservation.
